# Two-Dimensional Tungsten Disulfide-Based Ethylene Glycol Nanofluids: Stability, Thermal Conductivity, and Rheological Properties

**DOI:** 10.3390/nano10071340

**Published:** 2020-07-09

**Authors:** Syed Nadeem Abbas Shah, Syed Shahabuddin, Mohd Faizul Mohd Sabri, Mohd Faiz Mohd Salleh, Suhana Mohd Said, Khaled Mohamed Khedher, Nanthini Sridewi

**Affiliations:** 1 Department of Mechanical Engineering, Faculty of Engineering, University of Malaya, Kuala Lumpur 50603, Malaysia; engr.nadeem@uet.edu.pk; 2 Department of Mechanical Engineering (Main Campus Lahore), University of Engineering and Technology, Lahore 54890, Pakistan; 3 Department of Science, School of Technology, Pandit Deendayal Petroleum University, Knowledge Corridor, Raisan Village, Gandhinagar 382007, Gujarat, India; 4 Department of Electrical Engineering, Faculty of Engineering, University of Malaya, Kuala Lumpur 50603, Malaysia; faizsalleh@um.edu.my (M.F.M.S.); smsaid@um.edu.my (S.M.S.); 5 Department of Civil Engineering, College of Engineering, King Khalid University, Abha 61421, Saudi Arabia; kkhedher@kku.edu.sa; 6 Department of Civil Engineering, High Institute of Technological Studies, Mrezgua University Campus, Nabeul 8000, Tunisia; 7 Department of Maritime Science and Technology, Faculty of Defence Science and Technology, National Defence University of Malaysia, Kuala Lumpur 57000, Malaysia

**Keywords:** nanofluids, tungsten disulfide, dispersion stability, thermal conductivity, rheology, surfactants

## Abstract

Developing stable nanofluids and improving their thermo-physical properties are highly important in heat transfer applications. In the present work, the stability, thermal conductivity, and rheological properties of tungsten disulphide (WS_2_) nanoparticles (NPs) with ethylene glycol (EG) were profoundly examined using a particle size analyzer, zeta-sizer, thermal property analyzer, rheometer, and pH measuring system. WS_2_ NPs were characterized by various techniques, such as XRD (X-Ray Diffraction), FTIR (Fourier Transform Infrared Spectroscopy), FESEM (Field emission scanning electron microscopy), and high-resolution transmission electron microscopy (HRTEM). The nanofluids were obtained with the two-step method by employing three volume concentrations (0.005%, 0.01%, and 0.02%) of WS_2_. The influence of different surfactants (Sodium dodecyl sulphate (SDS), Sodium dodecylbenzenesulfonate (SDBS), Cetyltrimethylammonium bromide (CTAB)) with various volume concentrations (0.05–2%) on the measured properties has also been evaluated. Pristine WS_2_/EG nanofluids exhibit low zeta potential values, i.e., −7.9 mV, −9.3 mV, and −5 mV, corresponding to 0.005%, 0.01%, and 0.02% nanofluid, respectively. However, the zeta potential surpassed the threshold (±30 mV) and the maximum values reached of −52 mV, −45 mV, and 42 mV for SDS, SDBS, and CTAB-containing nanofluids. This showed the successful adsorption of surfactants onto WS_2_, which was also observed through the increased agglomerate size of up to 1720 nm. Concurrently, particularly for 0.05% SDS with 0.005% WS_2_, thermal conductivity was enhanced by up to 4.5%, with a corresponding decrease in viscosity of up to 10.5% in a temperature range of 25–70 °C, as compared to EG. Conversely, the viscoelastic analysis has indicated considerable yield stress due to the presence of surfactants, while the pristine nanofluids exhibited enhanced fluidity over the entire tested deformation range. The shear flow behavior showed a transition from a non-Newtonian to a Newtonian fluid at a low shear rate of 10 s^−1^. Besides this, the temperature sweep analysis has shown a viscosity reduction in a range of temperatures (25–70 °C), with an indication of a critical temperature limit. However, owing to an anomalous reduction in the dynamic viscosity of up to 10.5% and an enhancement in the thermal conductivity of up to 6.9%, WS_2_/EG nanofluids could be considered as a potential candidate for heat transfer applications.

## 1. Introduction

Various engineering applications—for instance, solar thermal, automobile engine cooling, and electronic cooling—employ heat exchanging systems to dissipate heat between two or more than two fluids [1,2]. The common heat transfer fluids used in many commercial heating and cooling processes include water, ethylene glycol, and oils [3,4]. Owing to their low thermal conductivity as compared to bulk metals, they exhibit poor heat dissipation capabilities. Since the development of nanofluids, these are presumed to be an alternative heat transfer carrier fluid. The thermo-physical properties of nanofluids, such as thermal conductivity and viscosity, play a vital role in improving the flow thermal system’s efficiency [5,6]. The viscosity is the internal resistance between the layers of fluids, which increases the pumping power [7]. In this context, the flow curve analysis can give sufficient information about the storage stability and pumping power required for the flow systems. Therefore, the rheological properties of nanofluids should be evaluated in the first instance [8]. Such rheological characteristics of nanofluids are attributed to the structural properties of the nanomaterials dispersed within the base fluid network [9]. Moreover, the thermal conductivity enhancement with the addition of nanoparticles (NPs) in the base fluid has been reported by many researchers, along with viscosity enhancement [10,11]. However, only a few studies are available in the literature which have reported a reduction in viscosity and an enhancement in thermal conductivity using colloidal solutions [12,13]. Therefore, a more rigorous investigation is required in order to identify the colloidal suspensions for flow thermal systems which not only improve thermal transport but also reduce the viscosity. The reduced viscosity can significantly mitigate the frictional losses during fluid flow. Thus, the reduced viscosity can add value in the efficiency improvement of flow thermal systems by decreasing the input pumping power.

As nanofluids have become next generation heat transfer fluids, with improved thermo-physical properties, many multifunctional nanomaterials have been utilized to exploit their potential in nanofluids. Currently, the two-dimensional, layered materials are of great concern for scientists and engineers due to their remarkable electrical, thermal, super-lubrication, optical, and catalytic properties [12,14,15,16]. In addition to graphene, transition metal chalcogenides (TMDCs) have attracted researchers due to their tremendous semiconducting and metallic properties, ascribed to their layered structure which is similar to that of graphene [15]. WS_2_ has shown better lubricity because of the weak intermolecular interactions among the sheets, which causes easy shearing with a fullerene-like structure [17,18]. Research has also focused on transition metal oxides in order to study their transport properties. Karimi-Nazarabad et al. have reported ~46.9% reduction in viscosity and 6.7% thermal conductivity enhancement using WO_3_-glycerol nanofluids, along with about two weeks of stability. In addition, an increase in viscosity for WO_3_-glycol and WO_3_-ethylene glycol nanofluids was also witnessed [8]. Recently, Kamel and Lezsovits demonstrated that WO_3_/water-based nanofluids improve the boiling heat transfer performance of horizontal heated copper tubes. However, a ~15% decline in the boiling heat transfer coefficient was observed as a result of nanoflake deposition on the wall of the tube, corresponding to a higher concentration. This suggests that the stability of the nanofluids is of great importance and should also be investigated parallel to the thermal and flow characteristics [19]. In addition, Paloma Martínez-Merino et al. have shown that the dispersion of MoS_2_ and WS_2_ increases the thermal conductivity of a typical concentrated solar plant (CSP) thermal oil. A maximum 45.6% and ~34.5% improvement in thermal conductivity was achieved corresponding to MoS_2_ nanowires and WS_2_ nanosheets, respectively, at an operating temperature of 90 °C [20]. Chen et al. have shown that the viscosity of EG-CNT nanofluids decreases at a particular concentration of carbon nanotube (CNT) due to the self-lubrication characteristics of CNTs [12]. In another report, transformer oil-based Ag-WO_3_ hybrid nanofluids have shown a 41% increase in thermal conductivity at a temperature of 100 °C [3]. Zhu et al.’s results revealed ~60.78% thermal conductivity enhancement using 0.75 vol% of CuO nanowires, along with a 6.41% increase in viscosity [21]. Pal et al. have elucidated up to 10% thermal conductivity improvement and three days of stability with a SiO_2_ coating over WO_3_ NPs in an aqueous phase. Moreover, the WO_3_ aqueous suspensions have displayed ~13.8% thermal conductivity enhancement without any coating material. This suggested that the coatings may reduce the thermal conductivity due to kapitza resistance at the solid–liquid interface [4]. Recently, two dimensional (2D) WS_2_ nanomaterial-based nanofluids have been characterized for concentrating solar power plants. It was reported that WS_2_ sheet incorporation did not alter the surface tension, viscosity, and specific heat capacity to a larger extent. However, the thermal conductivity improvement (~30%) reached a substantial value when compared to the typical heat transfer base fluid pertinent to CSP applications. There has also been observed no marked variation in the friction factor in comparison to the base fluid, suggesting no increase in pressure drop [22]. Additionally, the transport properties of nanofluids are also significantly affected by the shape, size, concentration of NPs, base fluid properties, pH, presence of surfactants, and the interface interaction between the base fluid and NPs [6]. Moreover, another long standing challenge for the commercial use of nanofluids is the colloidal instability [4,8]. The precipitation of colloidal suspensions is obvious due to the aging effect, which causes coagulation [23]. To re-disperse such coagulates is difficult in base fluids due to the high surface energy [24]. Consequently, it reduces the thermal conductivity of the nanofluids. Therefore, it has become necessary to prevent agglomeration and sedimentation by improving the surface charge of nanoparticles. Researchers have utilized various techniques, such as covalent (chemical structure modification) and non-covalent (using surfactants) functionalization, to improve surface activity. In addition, plasma functionalization has also been used to achieve high dispersion stability [25]. However, the process’ complexity and high cost still remains a limitation. Thus, surfactants are still an economical and facile choice to develop stable colloidal suspensions. Saterlie et al. reported the effect of surfactants (Polyvinylpyrrolidone (PVP), SDBS, oleic acid, and CTAB) on the thermal conductivity and dispersion stability of Cu-H_2_O colloidal suspensions. They found that the charge imbalance with time causes nanoparticle coagulation, which reduces the thermal conductivity [23].

The aforementioned studies have revealed that only a few experimental investigations have been carried out that are related to the stability and thermo-physical properties of TMDC-based nanofluids. However, the super-lubricity of TMDCs has the potential to improve the rheological properties of nanofluids, whereas the thermal, semiconducting, and metallic characteristics may also improve the thermal conductivity, leading to the better performance of heat transfer systems. In addition, to the best of the author’s knowledge, the literature has not reported much on the tungsten disulphide (WS_2_)/EG nanofluid’s stability, thermal conductivity, and rheological properties in the presence of different surfactants as stabilizers and rheology modifiers. As reported earlier, besides improvements in the dispersion stability, the surfactant can also reduce the viscosity. For instance, Zhou et al. have shown that the analogous micelle effect of SDS can lower the viscosity of water-based TiO_2_ nanofluids on top of the lubrication effect of nanoparticles [26]. As previously stated, SDBS and CTAB have not been explored in terms of their synergistic rheological property modifications. Therefore, in the present work, various surfactants, such as anionic (SDS, SDBS) and cationic (CTAB) surfactants, were chosen as an economical means for two reasons: firstly, to exploit their steric hindrance effect for the better dispersion of WS_2_/EG nanofluids [27]; secondly, the interfacial tension lowering characteristics may also contribute towards viscosity reduction [26]. Therefore, in the present work, a comprehensive experimental evaluation of WS_2_/EG nanofluids’ dispersion stability at 25 °C and the thermal conductivity in a particular temperature range (25–70 °C) has been carried out. The shear flow and viscoelastic behavior was evaluated at 25 °C, while the temperature sweep dynamic viscosity was studied between 25 and 70 °C. Furthermore, the influence of anionic (SDS, SDBS) and cationic (CTAB) surfactants on stability, thermal conductivity, and rheological properties have also been investigated. In addition, the rheological experimental results have also been correlated with suitable models to develop conceivable synergies. The objective of the present experimental study was to incorporate a novel 2D WS_2_ in EG-based nanofluids in order to explore their stability and thermo-physical properties for potential flow thermal systems.

## 2. Materials and Methods

### 2.1. Materials

All the materials in the experimental examinations were used without further modifications. The specifications of the nanoparticles and the surfactants and base fluid are shown in Table 1 and Table 2, respectively.

### 2.2. Methods

#### 2.2.1. Material Characterization Techniques

The phase identification and purity of the WS_2_ nanoparticles were carried out by observing the X-ray diffraction patterns using an Empyrean X-ray powder diffractometer (Malvern Panalytical Ltd., Malvern, UK) with Cu-Kα radiations with a wavelength of 1.54 Å over 2θ range 10–80°. Fourier transform infrared (FTIR) spectra were captured on a FT/IR-100 spectrophotometer (Perkin Elmer, Billerica, MA, USA) within the wavenumber range of 450–4000 cm^−1^ using KBr pellets. The morphology was confirmed by employing field emission scanning electron microscopy (FESEM, JSM-7600F, operated at 10 kV by JEOL Ltd., Tokyo, Japan) and high-resolution transmission electron microscopy (HRTEM, JEM-2100F by JEOL Ltd., Tokyo, Japan).

#### 2.2.2. Nanofluid Preparation

The WS_2_/EG nanofluids were prepared using a two-step method. The WS_2_ NPs were weighed corresponding to volume concentrations of 0.005% (0.375 mg/mL), 0.01% (0.7502 mg/mL), and 0.02% (1.5 mg/mL) and mixed with the base fluid along with various volume concentrations (0.05%, 0.5%, 1%, and 2%) of SDS, SDBS, and CTAB surfactants. The volume fraction of NPs and surfactants in liquid were obtained from the weight of the dry powder(s) and the total volume of the mixture. The mixture suspension was subjected to magnetic stirring (40 °C and 250 rpm) in order to break the agglomeration for 30 min. Finally, the colloidal suspension was sonicated (45 kHz) for 90 min to obtain homogeneous and stable nanofluids.

#### 2.2.3. Nanofluid Measurement Instruments/Techniques

The stability of the WS_2_/EG nanofluids was measured with the Litesizer 500 (Anton Paar, Graz, Austria) by estimating the zeta potential. It employs the electrophoretic light scattering (ELS) technique, as per ISO 13099-2:2012. The Litesizer 500 is also capable of measuring particle sizes within a range of 0.3 nm–10 µm with a minimum sample requirement of ~20µL. The measurement accuracy and repeatability for the reference material (polystyrene latex) is ~±10% and ~±2%, respectively. The particle size distribution was analyzed to determine the polydispersity index (PDI) of the solution and the hydrodynamic diameter of the particle(s). The particle size estimation is based on the dynamic light scattering technique, as per ISO 22412:2017. To estimate the particle size, supernatant liquid was used to dilute the samples. Supernatant liquid was prepared from the parent sample using a tabletop centrifuge (Anton Paar, Graz, Austria) at 5000 rpm for 3 min. The auto detection angle mode was used to avoid the effect of multiple scattering in the particle size measurements. However, the relative standard deviation of the particle size distribution for the three measurements was found to be less than 2%, with around 6% error. Furthermore, to correlate stability with the isoelectric point (IEP), the pH of all samples was measured using pH 700 (Thermo Fisher Scientific, Waltham, MA, USA). The calibration of the apparatus was done using buffer solutions with known pH of 4, 7, and 10. All measurements for stability evaluation were performed at 25 °C.

The thermal conductivity measurements of the WS_2_/EG nanofluids were conducted with a KD2 thermal conductivity meter (Decagon Devices Inc., Pullman, WA, USA), as per ASTM D5334 and IEEE 442-1981 [28]. The KS-1 sensor probe was used, with an accuracy of ±5% within 0.2–2 W/m.K [29]. Before starting measurements, the KS-1 sensor was calibrated with the standard glycerin solution, distilled water, and ethylene glycol. The calibration results were obtained within instrumental measurement uncertainty. The nanofluid thermal conductivity data were taken in a range of temperatures (25–70 °C). The samples were maintained at a constant temperature by employing the thermostat water bath (Daihan Scientific, Wonju-si, South Korea) for 30 min to minimize the influence of micro-convection on the measured results. In total, 10 measurements were recorded, and an average was given in the analysis. Finally, the uncertainty analysis of the thermal conductivity data showed that the standard deviation from the mean was less than ±5% and results were presented in the form (mean ± margin of error) with a 95% confidence interval. The absolute uncertainty of the thermal conductivity data is computed using Equation (1), considering a 95% confidence interval [30].

(1) $$x=\bar{x}\;\pm \;\frac{2.262\times \sigma }{\sqrt{n}}$$

$\bar{x}$ is the mean thermal conductivity, 2.262 is the factor taken from the *t*-distribution table (*t*-statistics) for the two-tail test against a 95% confidence interval, corresponding to a degree of freedom of nine, σ is the standard deviation of the measurement set, and *n* is the number of measurements.

Rheological measurements of the prepared WS_2_/EG nanofluids were carried out with the MCR series rheometer (Model 302-SN82171186, Anton Paar Asia Pacific Laboratory, Kuala Lumpur, Malaysia). The double-gap measuring geometry (DG 26.7-SN39066) with a 1 mm gap was chosen to test the low viscosity samples [31]. The small gaps between the concentric cylinders allow measurements with sample volumes as small as 3 mL. Before commencing the measurements, the motor torque was adjusted with a quick air check for reliable and repeatable measurements. In addition, the rheometer was calibrated with water and ethylene glycol. The maximum error was computed at ~5% in a temperature range of 10–70 °C, with ~4% relative standard deviation. Nevertheless, Yu et al. have already revealed the maximum errors to be ~3% and ~5% in rotational and oscillation measurements, respectively, for DG (double gap) measuring geometry [31]. To minimize the pre-shear history effects, samples were placed static inside the holder at constant temperature for 10 min. The shear flow behavior of the nanofluids was examined at 25 °C in a shear rate range (1–1000 s^−1^) in order to determine the dynamic viscosity and flow behavior. The temperature sweep test (25–70 °C) was performed at a constant shear rate (50 s^−1^ with pre-shear for 180 s), with a temperature ramp of 2 °C/min. Furthermore, the direct strain oscillation (DSO) test was conducted to study the structural behavior of the nanofluids (viscoelastic behavior) at a constant frequency (1 Hz) and variable strain/deformation (0.01–1000%). This test allows an insight into the nanofluids’ structural linearity in the form of the linear viscoelastic region (LVER), flow point, true yield point, G’ (storage modulus), and G” (loss modulus). Finally, the Rheo Compass software (V1.22.435, Anton Paar, Graz, Austria) was employed to record and analyze the rheological data.

## 3. Results

### 3.1. Material Characterization Analysis

**Figure 1:**
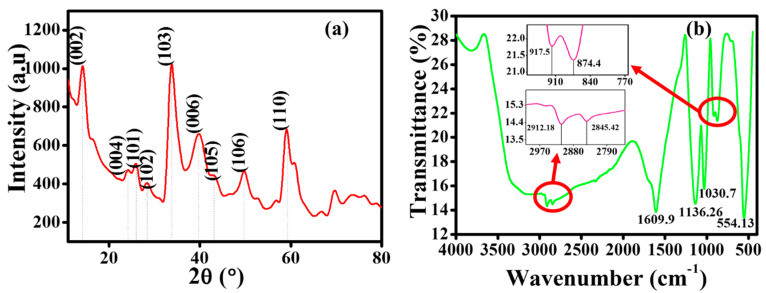
(**a**) Powder XRD pattern of WS_2_ and (**b**) Universal Attenuated Total Reflectance (UATR)-FTIR spectra of WS_2_.

XRD was performed to investigate the crystallinity of the WS_2_ nanostructure. Though it was challenging to utilize powder XRD for WS_2_, the diffraction pattern exhibited nine sufficiently resolved peaks. Figure 1a shows the XRD pattern of WS_2_ nano powder; the first peak appeared at 2θ ~14.3°, which has been ascribed to the (002) plane. This particular plane (002) represents the hexagonal structure of the WS_2_ [32]; it also indicates the multilayer structure of the WS_2_ nano powder [33]. The inter-plane distance (d-spacing) at 2θ ~14.3° was calculated as ~6 Å (using Bragg’s Law), which is typical for TMDCs [15,18]. The successive peaks were located at 2θ ~24.4° (004), 2θ ~25.9° (101), 2θ ~28.4° (102), 2θ ~33.4° (103), 2θ ~39.3° (006), 2θ ~43.2° (105), 2θ ~49.9° (106), and 2θ ~58.9° (110), with their corresponding reflection planes. These XRD peaks were well matched with 2H–WS_2_–JCPDS (Joint Committee on Powder Diffraction Standards) number (84–1398) [15]. Additionally, the presence of (002), (101), and (110) planes confirmed the multilayer 2D structure of WS_2_ nano powder [16].

**Figure 2:**
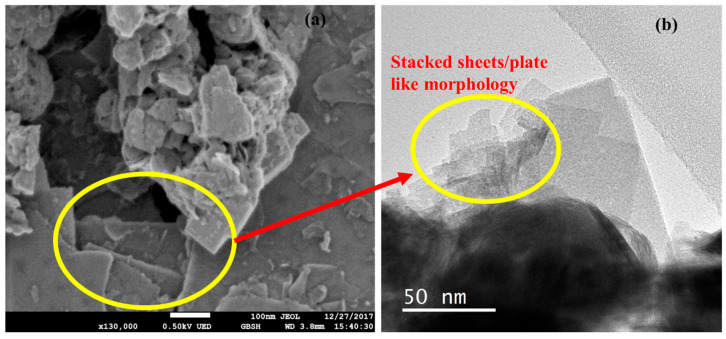
WS_2_ morphology using (**a**) FESEM and (**b**) HRTEM (high-resolution transmission electron microscopy).

Furthermore, the morphology of WS_2_, as obtained from FESEM and HRTEM, is shown in Figure 2. It can be seen from the morphology results that the WS_2_ nano powder has a sheet or plate-like structure [16]. The mean size was approximately 90 nm according to the supplier, which was well in agreement with the Debye–Scherrer calculations (~93 nm) [34]. Subsequently, the FTIR spectra were recorded to identify the chemical bonds in the molecules, with the assistance of infrared absorption bands. This analytical tool distinguishes the functional groups and offers an insight into the covalent bonds. Figure 1b clearly highlights the distinct peaks of the vibrational modes for WS_2_ nano powder. The peak that appeared at around 554.13 cm^−1^ was attributed to the W-S bond, whereas the one at around 917.5 cm^−1^ was ascribed to the S–S bond. On the other hand, the peak position at around 1609.9 cm^−1^ was referred to as the stretching deformation of the hydroxyl group, and 2912.18 cm^−1^ corresponded to the O–H vibration [18,35].

### 3.2. Stability and Particle Size Distribution Analysis

**Figure 3:**
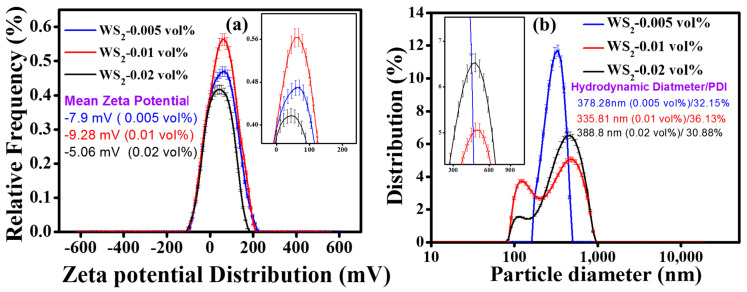
(**a**) Zeta potential distribution without dispersant and (**b**) particle size distribution without dispersant.

Figure 3a shows the zeta potential distribution of pristine WS_2_/EG nanofluids. The mean zeta potential was obtained to be −7.9 mV, −9.28 mV, −5.06 mV, corresponding to 0.005%, 0.01%, and 0.02% volume concentrations, respectively. Contrary to the zeta potential distribution, the particle size distribution in Figure 3b shows that the mean particle diameter was about 378.28 nm, 335.81 nm, and 388.8 nm, corresponding to mean zeta potential values of −7.9 mV, −9.28 mV, and −5.06 mV, respectively. Here, it is interesting to note that the particle size was well-linked with the zeta potential. For instance, the higher mean zeta potential corresponded to a smaller mean particle diameter. Nevertheless, at the moment, this was the case of pristine nanofluids (without surfactant) only. However, the particle size distribution at higher concentrations (0.01 and 0.02 vol%) of WS_2_ showed bi-modal peaks, which indicated the agglomeration tendency of colloids, leading to different aggregate sizes in the WS_2_/EG nanofluids. In fact, the Van der Waals interaction among the co-particles caused this behavior, as the higher concentration reduced the inter-particle spacing. All in all, the zeta potential and particle size distribution suggested the optimum concentration of WS_2_/EG nanofluids, as the 0.005% volume concentration of pristine WS_2_/EG nanofluids showed intermediate zeta potential (−7.9 mV) and a unimodal particle size distribution peak of around 378.28 nm. Thus, this can be regarded as the optimum concentration, with no significant tendency of agglomeration, even though 0.01 vol% nanofluid showed a high mean zeta potential (−9.28 mV) but bi-modal distribution peaks. Such observations have also been reported in the literature, where the mean particle size decreased as the zeta potential increased [36]. Previous investigations on nanofluids have demonstrated a threshold value of zeta potential (>±30 mV), for which nanofluids are regarded as stable [37,38]. In the current work, three different surfactants, such as SDS, SDBS, and CTAB were added in order to surpass the zeta potential threshold.

#### Effect of Surfactants on Stability of WS2/EG Nanofluids

**Figure 4:**
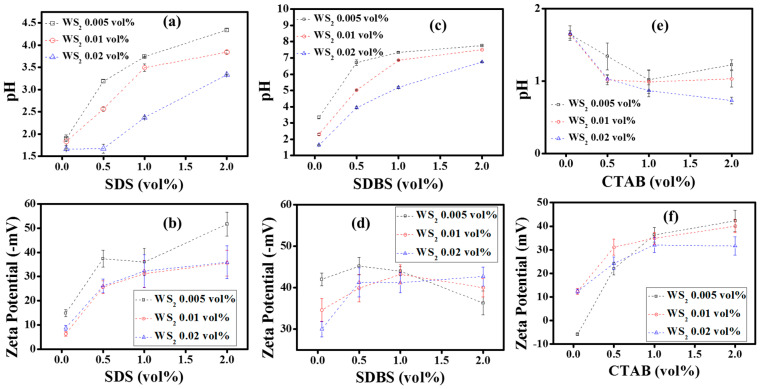
Relationship between pH and zeta potential at different volumes% of WS_2_ and dispersants (**a**,**b**), with SDS, (**c**,**d**) with SDBS, and (**e**,**f**) with CTAB.

Figure 4 shows the influence of surfactants on the mean zeta potential and pH of WS_2_/EG nanofluids. It can be observed from Figure 4b that, in contrast to pristine WS_2_/EG nanofluids, the SDS surfactant showed an increasing trend of mean zeta potential with concentration dependency. The mean zeta potential value shifted from −14.9 to −51.7 mV for 0.005% of WS_2_/EG nanofluid as the SDS surfactant concentration varied from 0.05 to 2 vol%. Similar behavior of SDS with 0.01% and 0.02% Ws_2_/EG nanofluids was also observed, but only the higher concentrations (1 and 2 vol%) of SDS crossed the threshold limit of mean zeta potential. On the other hand, SDBS’ response to WS_2_/EG nanofluids was rather interesting, as the mean zeta potential exceeded the threshold limit for all concentrations of nanofluids, as shown in Figure 4d. This may be due to the fact that the aromatic characteristics of SDBS have a strong tendency towards steric hindrance for better colloidal dispersion [38]. However, the CTAB surfactant’s interaction with the WS_2_/EG nanofluids was quite similar to that of SDS, but the zeta potential inversion (negative to positive) became significant at small concentrations of nanofluids, as shown in Figure 4f. A summary of the maximum mean zeta potential corresponding to the optimum surfactant concentrations for all nanofluid concentrations is shown in Table 3. These high zeta potentials were attributed to the surfactant layers being adsorbed onto the particle’s surface, which weakens the Van der Waals forces and prevents aggregation. Moreover, the stability of nanofluids is also dependent on the pH value, because the magnitude of the surface charge is also indicated by the pH value of the colloidal system [39]. Therefore, the pH of all samples was measured and correlated with the zeta potential, as shown in Figure 4a,c,e. The surfactant’s addition showed an exponential increasing trend in pH value with concentration, which indicated a gradual increase in the surface charge. This increasing trend in pH was found to be proportional to the mean zeta potential in a similar manner for most of the nanofluids which contained surfactants.

**Figure 5:**
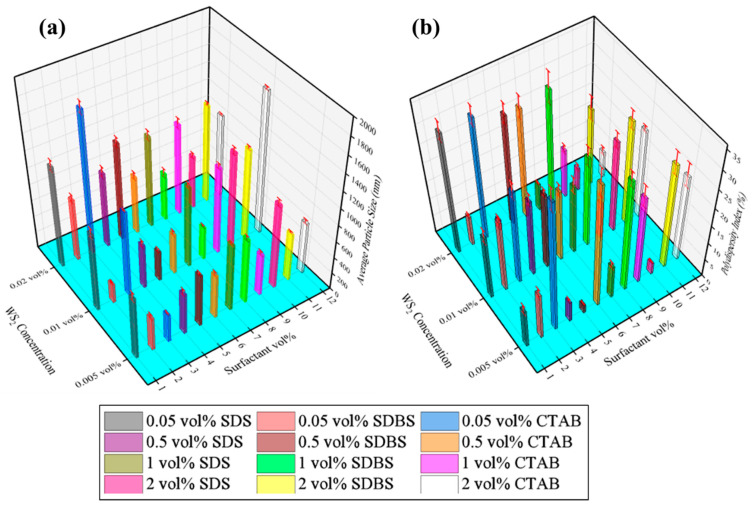
(**a**) Effect of surfactants on mean particle size and (**b**) polydispersity index (PDI) of WS_2_/EG nanofluids.

Apart from the mean zeta potential enhancement due to the steric hindrance effect, the increase in particle size was also noticeable. This size increment was attributed to the adsorption of surfactant molecules onto the particles’ surfaces, as shown in Figure 5a. Furthermore, the particle size distribution did not show much influence due to the surfactant’s adsorption, as the polydispersity index (PDI) was found to be sufficiently below 30, indicating the mono-dispersed state of most of the tested samples, as shown in Figure 5b. This means that the adsorption of surfactants increases the particle size but is less likely to form different sized aggregates in the WS_2_/EG nanofluids. However, PDI depends on the morphology of particles as well, and, according to ISO 22412:17, any value <7% is regarded as having a mono-dispersed state for spherical shaped particles. However, in the present case, the morphology was sheet-like, as shown in Figure 2. Therefore, it could be postulated that any PDI value <30% can be regarded as a mono-dispersed state for the non-spherical particles.

The WS_2_/EG nanofluids remained mono-dispersed for most of the formulations, and all the tested samples showed a secondary minimum potential energy behavior, according to DLVO theory. This means that the aging effect may cause the weak agglomeration of dispersions, which can be re-dispersed with an external agitation mechanism. Here, it is also worth mentioning that the density of particles is higher than the base fluid, as presented in the Materials section. This might cause the sedimentation of particles with time under the influence of gravity, irrespective of the type of surfactant. Nonetheless, the long-term colloidal instability of nanofluids is an unresolved mystery, as the aging effect is obvious from the present work. Consequently, a continuous investigation of colloidal stability is on its way as a recent development has introduced a novel three-step interface-based method to improve the stability of nanofluids. The newly developed technique works on the balance of the polar and dispersive components of the base fluid, which is well-suited to nanomaterial dispersion. Such adjustment leads to a minimum tension at the solid–liquid interface by using the non-ionic surfactant Triton X-100 (Panreac Quimica SLU, Barcelona, Spain). This approach showed better stability and an anomalous enhancement in thermo-physical properties based on the molecular dynamic simulation, even at 70 °C [40]. Meanwhile, in another recent report, Li et al. have shown the spacer length optimization of cationic Gemini surfactant that improved the colloidal stability of aqua-based Au and Ag nanofluids, along with the pH dependency of stability. It was also noted that at high temperatures, the bilayer coating of the surfactant becomes looser, which results in the poor stability of the nanofluids due to less surface charge density [41]. Conclusively, the long-term colloidal stability is presumed to be a crucial factor in determining the improvement in the thermo-physical properties of nanofluids.

As demonstrated by Liu et al., the convective heat transfer coefficient is strongly related to the particle size and dispersion stability [36]. Among many techniques, zeta potential is commonly used in the estimation of a nanofluid’s colloidal stability. It estimates the surface charge between the dispersed phase (nanoparticles) and continuous phase (base fluid). This surface charge determines how strong the repulsive force is that exists among the dispersed phase for long-term stability [42]. Conversely, the Van der Waals forces attract the particles, leading to agglomeration. This phenomenon is described by the colloidal theory (DLVO theory). The DLVO theory is an archetype to comprehend the balance between the Van der Waals and electrostatic forces, which determines the colloidal stability. On the other hand, the robust Van der Waals forces on the nanoparticle’s surface tries to attract other co-particles, which causes aggregation [43]. As a result, the large agglomerates/clusters undergo quick sedimentation under the influence of gravity. This instability owing to agglomeration can cause chocking in micro-channels and can decrease the thermal performance of nanofluids. Therefore, the understanding of the interfacial interactive forces (particle–liquid) are of considerable importance regarding the stability of nanofluids [44]. Generally, the colloidal stability is controlled by the pH value, surfactant, temperature, and viscosity of the nanofluid [41]. Therefore, it can be concluded from the findings of the present work that the addition of surfactants has sufficiently improved the mean zeta potential and the pH value, but it has also increased the aggregate size of the colloids.

More precisely, for 0.005% WS_2_, the addition of SDS (0.05%) increased zeta potential by ~88% in comparison to pristine nanofluid. This rate of improvement was reduced to 37% per 0.05% SDS addition when 0.5% SDS was incorporated, but the absolute value of the zeta potential increased. Similarly, for other nanofluid combinations with SDS, the absolute zeta potential values improved, but the rate of improvement with regards to surfactant concentrations became slow as the amount of surfactant increased. This also suggests that analogous micelles have been formed in the nanofluid formulations, which could be visualized in the form of increased agglomerate size as well. In case of SDS addition, the maximum increments in agglomerate size appeared at ~172%, ~245%, and 261%, corresponding to 0.005% WS_2_ + 2% SDS, 0.01% WS_2_ + 2% SDS, and 0.02% WS_2_ + 0.05% SDS, respectively. On the other hand, 0.05% SDBS increased the zeta potential to 431% for 0.005% WS_2_ nanofluid. Subsequently, the rate of increment was reduced to 47% per 0.05% SDS addition when 0.5% SDBS was added. At the same time, the maximum enhancement in agglomerate size was observed at ~108%, 233%, and 254%, corresponding to 0.005% WS_2_ + 1% SDBS, 0.01% WS_2_ + 2% SDBS, and 0.02% WS_2_ + 2% SDBS, respectively. However, for 0.05% CTAB, no significant variation in zeta potential was observed for 0.005% WS_2_ nanofluid, but for higher concentrations of CTAB, beyond 0.5%, the maximum enhancement was observed to be ~178%. Like SDS and SDBS, the enhancement rate was reduced to 18% per 0.05% CTAB when the concentration of CTAB was increased to 1%. In addition, the agglomerate size grew to ~67%, 410%, and 408%, corresponding to 0.005% WS_2_ + 2% CTAB, 0.01% WS_2_ + 2% CTAB, and 0.02% WS_2_ + 0.05% CTAB, respectively.

### 3.3. Thermal Conductivity Analysis

**Figure 6:**
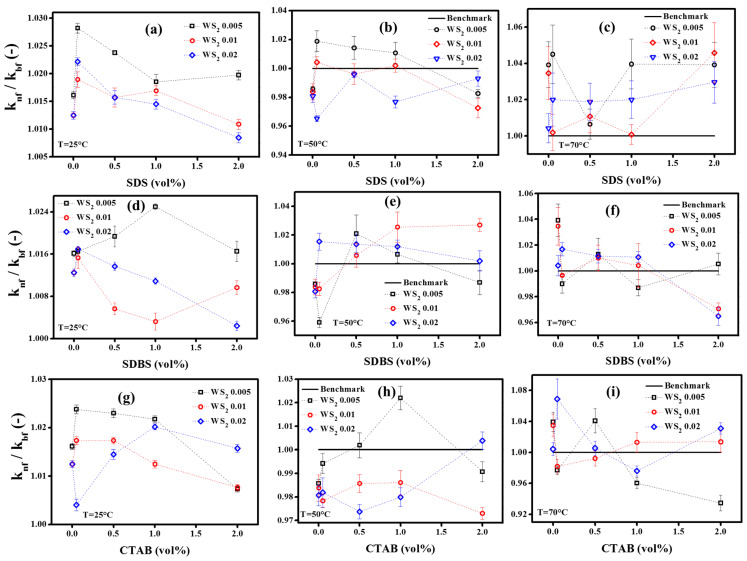
Relative thermal conductivity at different temperatures: (**a**–**c**) with SDS, (**d**–**f**) with SDBS, and (**g**–**i**) with CTAB.

Based on the stability evaluation, the prepared samples have shown a reversible nature. Therefore, to deeply understand their potential towards thermal performance enhancement, all samples were tested and compared for thermal conductivity. The mean thermal conductivity data of the WS_2_/EG nanofluids, based on the uncertainty analysis with a 95% confidence level, is presented in Table S1 (Supplementary Materials). Meanwhile, Figure 6 shows the relationship between the relative thermal conductivity (*k_nf_*/*k_bf_*) of WS_2_/EG nanofluids and surfactant concentration over different temperatures. Initially, the relative thermal conductivity of pristine WS_2_/EG nanofluids was computed from the mean thermal conductivity data and compared with the surfactant-containing nanofluids.

Figure 6a–c represents the effect of SDS surfactant on relative thermal conductivity at 25 °C, 50 °C, and 70 °C, respectively. At 25 °C, the relative thermal conductivity increased with the addition of SDS, which was found to be a maximum of ~2.8% more when compared to the base fluid, corresponding to a minimum volume concentration of SDS (0.05 vol%). Moreover, the maximum thermal conductivity enhancement for SDS-containing nanofluids was observed to be around 2.8%, 1.9%, and 2.2%, corresponding to 0.005%, 0.01%, and 0.02% WS_2_, respectively. Meanwhile, the corresponding improvements without surfactants were 1.6% (0.005% nanofluids) and 1.2% (0.01 and 0.02% nanofluids). Furthermore, the results revealed that any successive volume concentration of SDS beyond 0.05% also improved the thermal conductivity, but it had a lesser effect when compared to the former. Such a thermal conductivity enhancement trend was noted for the 0.005% and 0.01% nanofluids, while 0.02% nanofluids showed a decrease in thermal conductivity at a 2% SDS volume concentration. This may be attributed to the fact that the higher concentration of surfactant adsorbed onto the particle’s surface may offer additional resistance to heat conduction. Consequently, a decrease in thermal conductivity was observed at a higher concentration of surfactant. However, at elevated temperatures, the behavior of the nanofluids was interesting. For instance, at 50 °C, the performance of the nanofluids decreased significantly, except for 0.005% nanofluids with SDS volume concentrations of up to 1%. However, the overall thermal conductivity improvement was significant when compared to the base fluid. On the other hand, the pristine nanofluids showed thermal conductivity reductions of about 1.4%, 1.6%, and 1.9%, corresponding to 0.005%, 0.01%, and 0.02% nanofluids, respectively, as compared to the base fluid. Such behavior at a temperature of 50 °C can be ascribed to the possible flocculation of aggregates under the influence of gravity due to the reduction in the buoyancy force in case of pristine nanofluids. The temperature rise is supposed to intensify the Brownian motion, which results in more collision as well as a reduction in the viscosity. Besides this, at a higher temperature, the surfactant adsorption might start dissociating, leading to aggregation and instability, which affects the thermal performance [45]. In contrast to pristine nanofluids, the surfactant induced steric effect competes to balance the gravity effect and suppresses the quick flocculation up to certain concentrations (of SDS) beyond which flocculation might happen. This might decrease the thermal conductivity for any successive SDS concentration. Therefore, the SDS-containing nanofluids showed a thermal conductivity enhancement for certain concentrations. However, interestingly, at a temperature of 70 °C, the pristine nanofluids again showed significant improvements in thermal conductivity up to values of 3.9%, 3.5%, and 0.4%, corresponding to 0.005%, 0.01%, and 0.02% of WS_2_, respectively. Possibly, this intriguing behavior occurred due to the enhanced Brownian motion of the suspended, small-sized particles within the base fluid network at 70 °C. In addition to pristine nanofluids, SDS-containing formulations also showed thermal conductivity enhancements of about 4.5%, 4.6%, and 3%, corresponding to 0.005%, 0.01%, and 0.02% nanofluids, respectively, at certain SDS concentrations of 0.05%, 1%, and 2%, respectively. The possible reason for this enhancement at the elevated temperature of 70 °C could be the Brownian motion and the steric stabilization of small-sized colloids in the case of SDS-containing nanofluids. However, a fundamental investigation is further proposed in order to more deeply understand the physical mechanism behind this behavior of nanofluids. Similarly, the addition of SDBS and CTAB also reflected a synergistic improvement in the thermal conductivity of WS_2_/EG nanofluids, which also showed a concentration dependency, as depicted in Figure 6d–i, respectively. Thus, due to the concentration dependency, an oscillatory behavior was observed for the nanofluids, which suggested the optimized formulations that might influence the thermal conductivity the most.

Here, it is also noteworthy that the present data show similar behavior to those reported by Xia et al. The reason behind the oscillatory behavior of the tested samples against surfactant concentration was the critical micelle concentration (CMC). The lower surfactant amount may provide an insufficient steric effect, and a higher concentration may cause flocculation, leading to coagulation and sedimentation. Under such conditions, the thermal performance of nanofluids may be reduced [46]. Another plausible reason for the deterioration of thermal performance at an elevated temperature is related to the foaming formation due to the presence of surfactants [47]. Concurrently, the influence of buoyancy forces cannot be overlooked regarding the long-term stability of colloidal systems, especially at elevated temperatures. The elevated temperature weakens the inter-layer friction forces of nanofluids which assist in increasing the aggregates’ terminal velocity under the influence of gravity. This happens due to reduced viscosity, density, and buoyancy forces [48]. Therefore, the colloidal system becomes unstable, which might affect the thermal performance of nanofluids. In addition, many thermal conductivity enhancement mechanisms have been demonstrated in previous studies, such as percolation networks, clustering, interfacial liquid layering, and the Brownian motion [49]. However, the accurate measurement of thermal conductivity also requires better stability, specifically at elevated temperatures. In this context, Sooraj et al. have demonstrated that PVA surfactant-coated CuO nanoparticles showed small crystallite sizes, which improved their stability as well as their thermal conductivity [50]. Recently, Ebrahimi et al. have proposed the use of gelling agents in order to prevent the micro-convection effect during the thermal conductivity measurement of nanofluids. They showed better measurement accuracy with this approach over a range of temperatures [51]. Likewise, a molecular dynamic simulation investigation of the Triton X-100 surfactant has also shown ~79.5% enhancement in thermal conductivity for aqua-based carbon nanotube nanofluids at 70 °C. This enhancement was attributed to the better colloidal stability with the inclusion of a surfactant which lowers the interfacial tension between the solid–liquid network [40].

Unlike the current investigation, previous studies have not shown any significant temperature dependence of nanofluids relative to thermal conductivity in the presence of surfactants. However, the increase in the absolute thermal conductivity of nanofluids has been referred to as the intrinsic behavior of base fluids [45]. In addition, it has also been reported that the addition of surfactants into the base fluid network may affect the thermal conductivity and viscosity [47]. To gain insights into such behaviors, all the studied surfactants at targeted concentrations and temperatures were tested for thermal conductivity (Figure S1 Supplementary Materials). The results revealed that the surfactants’ inclusion reduced the thermal conductivity of the base fluid at all targeted concentrations and temperatures, except for the 25 °C temperature. A similar effect of surfactants on a base fluid was also reported in the literature [52]. Moreover, it was also observed that the samples with high values of zeta potential did not show more thermal conductivity enhancement. This may be due to the utilization of high concentrations of surfactants to induce the steric hindrance effect. On one hand, the high concentration improved the zeta potential, as discussed earlier, but, on the other hand, the high concentration also mitigates thermal conduction at the solid–liquid interface. This particular thermal conduction hindrance is termed the kapitza resistance. Such behavior of nanofluids was also reported in a previous study related to nanofluids, conducted by Leong et al. [53]. Contrary to the present and investigation and that of Leong at al., Liu et al. showed that small-sized particles are not necessary to show better thermal performance. Instead, it is the stability of the colloidal system in terms of the mean zeta potential and the mean particle size which determines the thermal performance enhancement of nanofluid systems [36].

In summary, it could be postulated that the WS_2_/EG nanofluids did not show any consistent trend of thermal conductivity based on concentration and temperature. It has to be noted that there is no study related to WS_2_/EG nanofluids reported in the literature to which we can compare the current data. However, an improvement in thermal conductivity was observed with surfactant addition, due to the better dispersion stability [45]. Therefore, for a detailed comparison, more rigorous experimental work is proposed in order to build the database before drawing a final conclusion. Most importantly, the stability should be studied quantitatively at higher temperatures to further understand the behavior of thermal conductivity.

Furthermore, the quantitative results can be summarized as follows: the maximum thermal conductivity enhancements were observed to be ~2.8%, 1.9%, and 4.5% for the combination of 0.05% SDS + 0.005% WS_2_, corresponding to operating temperatures of 25 °C, 50 °C, and 70 °C, respectively. Subsequently, the increased concentration of SDS decreased the thermal conductivity, which is a common observation for higher concentrations of surfactants. Like 0.005% WS_2_, the higher concentrations, such as 0.01% WS_2_ and 0.02% WS_2_, also showed higher enhancement corresponding to lower surfactant concentrations. However, the elevated temperature behavior showed an oscillating response and the reason for this may lie within the detachment of surfactant molecules and their interaction with the WS_2_ sheets. Consequently, this needs to be explored further. Therefore, the current work contributes to the production of optimum WS_2_/EG nanofluid formulations that have improved thermal conductivity and stability. Hence, the optimum nanofluid formulations in terms of improved thermal conductivity that were obtained as a result of the current research work are shown in Table 4.

### 3.4. Rheological Analysis

The rheological analysis was carried out based on the optimized samples obtained from the thermal conductivity analysis and compared with the pristine WS_2_/EG nanofluids. In total, 12 samples were selected from three groups (SDS, SDBS, and CTAB) of nanofluids based on the maximum thermal conductivity enhancement. The selected nanofluids were then subjected to oscillation and rotational rheological measurements to gain an insight into their structural and flow behavior.

#### 3.4.1. Oscillation Measurements (Strain Sweep Test)

**Figure 7:**
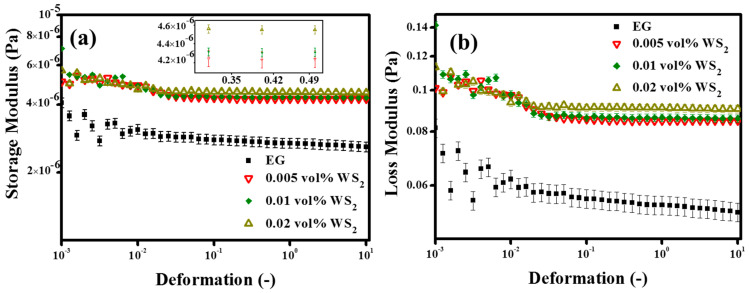
(**a**,**b**) Storage (G’) and loss (G”) modulus variation in WS_2_/EG nanofluids without surfactants.

In flow thermal systems, the viscous nature dominates while the fluid is flowing, but sometimes the nature of the fluid alters due to structural changes, which may put an additional load on the system in terms of pumping power. Although, EG is known to be the Newtonian fluid, the addition of nanoparticles might change its nature from viscous to elastic [54]. Therefore, to predict and analyze the structural changes in nanofluids in the presence of nanoparticles and surfactants, an oscillation strain sweep test was performed. In fact, the strain sweep test is very sensitive and can give upfront information about the structural changes in nanofluids. Figure 7a,b represents the storage (G’) and loss modulus (G”) for pristine WS_2_/EG nanofluids at the tested volume concentrations (0.005%, 0.01%, 0.02%). It can be seen from the results shown in Figure 7a,b that the storage modulus (G’) was lower compared to the loss modulus (G”) over the entire deformation range for EG as well as for the pristine WS_2_/EG nanofluids. Thus, the oscillatory strain sweep results verify the pure viscous nature of the EG base fluid. However, it is noticeable from the figure that the addition of the WS_2_ NPs significantly changed both G’ and G”, but still the viscous nature remained prominent. Overall, the loss factor (G”/G’) was very high, indicating the pure viscous flow behavior of pristine WS_2_/EG nanofluids over the entire tested deformation range. These results confirmed the super-fluidity of the pristine WS_2_/EG nanofluids.

**Figure 8:**
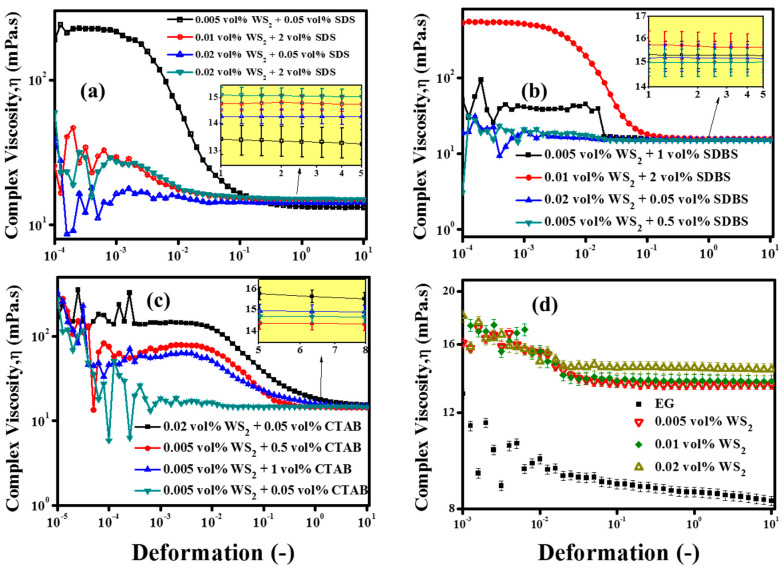
(**a**–**c**) Complex viscosity (*η*) of WS_2_/EG nanofluids with surfactants and (**d**) without surfactants as an indication of structured network inclusion.

Apart from the loss factor, the complex viscosity (*η*) was also an indicator for the structural changes in the nanofluids due to the addition of NPs. As shown in Figure 8d, the NPs’ addition increased the complex viscosity (*η*) as compared to the EG base fluid. Like the pristine WS_2_/EG nanofluids, surfactant-containing nanofluids also showed a substantial variation in *η*, which indicated the structural changes in the nanofluids, as shown in Figure 8a–c. The highest *η* means that the nanofluids may require more input energy to initiate the motion of nanofluids as compared to the EG base fluid. Furthermore, the pristine nanofluids showed a concentration dependency on the structured network. It can be seen from the figure that the elastic nature of the pristine nanofluids increased with the concentration of WS_2_ NPs. This means that the higher the concentration, the higher the pumping pressure that will be required to initiate the flow. Besides this, the surfactant-containing nanofluids transformed the structural arrangement of nanofluids and shifted it to the highest elastic domain.

**Figure 9:**
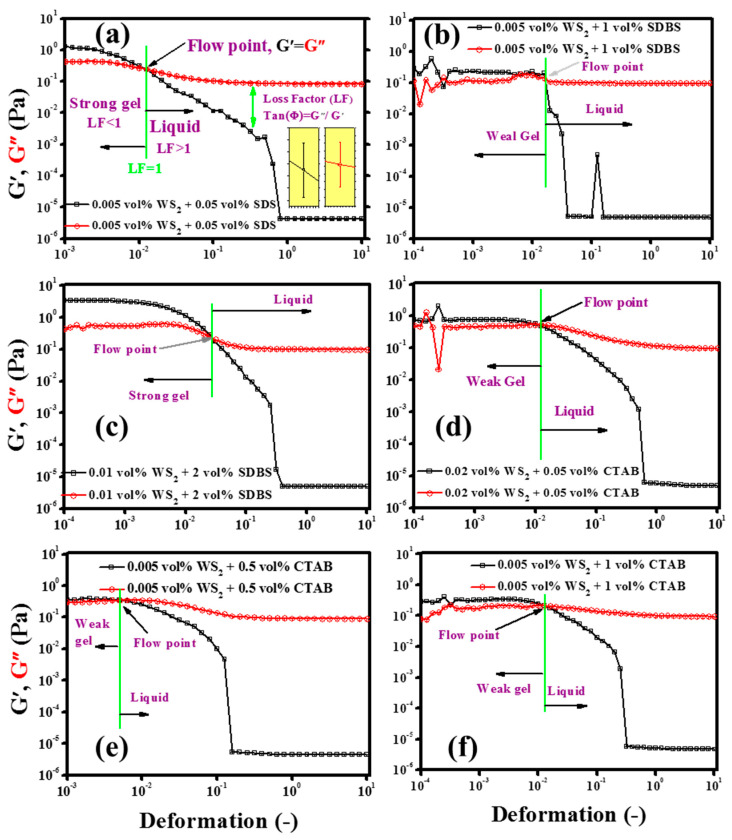
Effect of surfactants on the viscoelastic behavior of WS2/EG nanofluids: evidence of typical flow point. (**a**) WS_2_ combination with SDS, (**b**,**c**) WS_2_ combination with SDBS, and (**d**–**f**) WS_2_ combination with CTAB.

As Figure 9 displays, the surfactant-containing nanofluids showed a hallmarked plateau of storage modulus (G’) over the loss modulus (G”), which highlighted the dependency on the type and concentration of surfactant as well as on the interaction between surfactants and NPs. Possibly, this was due to the complex formations which occurred because of the physical interactions between the NPs and surfactant molecules in the nanofluids. As shown in Figure 9, the storage (G’) and loss (G”) modulus were parallel to each other over a certain range of deformation. This region is referred to as the linear viscoelastic range (LVER). In this region, the nanofluid behaves like a gel, with a pronounced elastic modulus, and the stress value that corresponds to the storage (G’) modulus in this limiting region is referred to as the true static yield stress [55]. It can also be observed from Figure 9 that, after a certain value of deformation, the structure breakdown started, followed by the nanofluid flow. The intersection of the storage (G’) and loss (G”) modulus corresponded to the flow point and the stress value mimicked the minimum force needed to initiate the flow of the colloidal suspension. Before the flow point, the stress value could not initiate the flow, which highlighted the existence of a critical stress value, and the fluid behavior was fully elastic. Beyond the flow point, the viscous nature became dominant, which indicated the liquid-like behavior of the nanofluids [56]. The similar phenomenon of structured nanofluids has also been observed in previous studies for exfoliated graphite nanoplatelets and titanium nitride-based EG nanofluids [54,55].

Furthermore, our results revealed that the overall behavior of nanofluids was highly dependent on the loss factor (LF). A low LF value gave rise to the gel-like behavior, which must be avoided in the case of flow thermal systems. Therefore, a high LF is needed so that the pumping power can be controlled to be as minimal as possible. In short, the strain sweep test could be exploited for the nanofluid’s preliminary structural evaluation. However, a detailed oscillation investigation is needed for samples which show a clear LVER state with very low LF value. In such cases, usually, frequency sweep and 3ITT tests are performed within the LVER range. In the present study, only a few samples showed an LVER state but without a low LF value. Therefore, the study was kept limited to the strain sweep test in order to gain an insight into the nanofluids’ structural behavior. In addition to the viscoelastic structure information of nanofluids, the strain sweep test also gives insights about the colloidal systems’ interactive forces, which determine the stability. The sterically stabilized colloidal systems have typical critical strain values of ~1% to 5% [56]. In the present study, surfactant-containing nanofluids have shown such a critical strain range. However, low critical strain values were also observed for some nanofluid formulations, probably due to weak colloidal stability. The results of nanofluid formulations which did not show a distinct LVER state are shown in Figures S2–S4 (Supplementary Materials). Thus, in the present work, for the tested nanofluids, the viscous flow behavior prevailed over the entire deformation range, along with the indication of a weak elastic nature in some cases. A detailed summary of the oscillation evaluation of the nanofluids is presented in Table 5.

#### 3.4.2. Rotational Measurements (Shear Flow Behavior)

**Figure 10:**
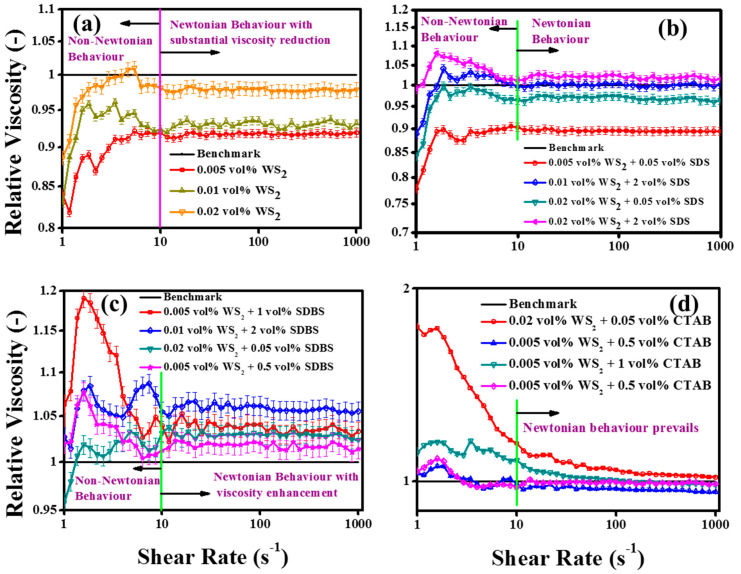
Relative viscosity of WS_2_/EG nanofluids (**a**) without surfactant and (**b**–**d**) with surfactants over a range of shear rates at 25 °C.

As the pumping flow may cause a variety of shear rates, the pumping performance of nanofluids was also evaluated with the rotational rheology by analyzing the shear flow behavior [56]. The base fluid dynamic viscosity was measured and found to be independent of shear rates between 10 and 1000 s^−1^. The pristine and surfactant-containing WS_2_/EG nanofluids’ viscosities are presented in terms of relative viscosity in Figure 10a–d, respectively.

The results revealed that all the nanofluids showed transformation from non-Newtonian to Newtonian behavior at a shear rate of 10 s^−1^. As shown in Figure 10a, the pristine nanofluids showed a substantial dynamic viscosity reduction with the addition of WS_2_ nanoparticles as compared to the base fluid (EG). Interestingly, the low concentration of WS_2_ showed the maximum viscosity reduction, followed by concentration dependent increments with higher concentrations (>0.005 vol%). These results were well in agreement with the strain sweep results, as the pristine nanofluids showed super-fluidity with high LF values. The lubricating effect of the NPs’ addition into the base fluid was witnessed. The NPs’ inclusions between base fluid layers caused the easy movement of the base fluid layers, which reduced the viscosity at all targeted temperatures and concentrations [57]. On the other hand, the SDS surfactant-containing nanofluids in the present work have also shown concentration dependency (Figure 10b). The small concentration of SDS did not much alter the dynamic viscosity, whereas the higher concentration increased the viscosity due to the formation of complexes. Unlike the SDS surfactant, the SDBS (Figure 10c) and CTAB (Figure 10d) surfactants significantly increased the viscosity of nanofluids, even at small concentrations, as compared to the pristine nanofluids, with the exception of 0.5 vol% of CTAB. Such distinct observations of dynamic viscosity can be attributed to physical structural modifications and the interfacial interaction of colloids [58].

As in a low shear rate regime, the colloids may be relatively unstable, and they may orient themselves in the flow direction at a high shear rate regime along with uniform distribution, with the breakdown of the large agglomerates. This might also cause fluctuations in the measuring system’s monitoring and recording capability of the force experienced by the spindle. Therefore, non-Newtonian behavior dominated at low shear rates (<10 s^−1^). More recently, such observations have been made that the hydrodynamic slip effects become more prominent at higher shear rates. Consequently, the nanosheets within the colloidal dispersions align themselves in the flow direction [59]. Therefore, together with nanosheet alignment in the flow direction, the lubricity of WS_2_ contributed to lowering the dynamic viscosity by minimizing the interfacial tension, as evident by the pristine nanofluids’ rheology. Thus, the hypothesis of the current research work came true as the anti-friction properties of WS_2_ were reflected well in the nanofluids in the form of reduced viscosity. Moreover, the synergistic effect of small concentrations of the SDS surfactant with WS_2_ NPs was also observed and found to be well in agreement with the results reported by Zhou et al. [26]. The pristine nanofluids showed ~8.2% less viscosity when compared with the EG base fluid, while with the addition of 0.05 vol% SDS, it was further reduced to ~10.5% for the 0.005% WS_2_/EG nanofluids. Such a viscosity reduction phenomenon may be attributed to the lubricating effect of NPs and analogous micelles of SDS surfactant. The present results revealed that the addition of the surfactant might significantly change the nature of the fluid, as the high concentrations of surfactants showed a viscosity enhancement effect. Like the oscillation evaluation of the nanofluids, the shear flow behavior also indicated similar rheology behavior in the presence of surfactants. Eventually, the oscillation and rotational measurements detected the structural variation with NPs and surfactant concentrations. Therefore, to compute the yield stress for structured fluids, many models have been proposed, such as Herschel–Bulkley (H–B) [54] and Bingham [55].

Equations (2) and (3) represent the H–B and Bingham models, respectively.

(2) $$\tau =\tau_{o}+K{\dot{\gamma }}^{n}$$

(3) $$\tau =\tau_{o}+\mu \dot{\gamma }$$

where *τ*, *τ_o_*, *μ*,$\;\dot{\gamma }$, *K*, and *n* are the shear stress, yield stress, Newtonian viscosity, shear rate, consistency, and the flow index, respectively. The experimental data of the measured samples were fitted with the above-mentioned models, and details of the flow parameters are given in Table 6. The model fitting results of yield stress were in good agreement with the strain sweep measurements for most of the samples, which showed significant concentration dependency. However, the Bingham model fit most of the tested samples well, compared to H–B. It can be seen from Table 6 that, for the Bingham model, the *τ_o_* value for EG was ~1.08, which reduced with the addition of NPs. This could be a rational indication of the super-lubricity of the pristine WS_2_/EG nanofluids, as observed in the dynamic viscosity measurements.

#### 3.4.3. Temperature Sweep Study

**Figure 11:**
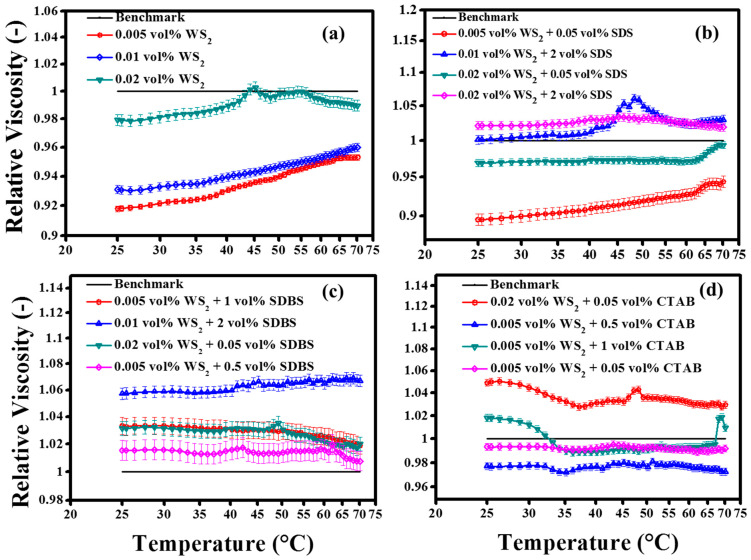
Effect of temperature on relative viscosity of WS_2_/EG nanofluids at 50 s^−1^ constant pre-shear: (**a**) pristine WS_2_/EG nanofluids, (**b**) SDS contained WS_2_/EG nanofluids, (**c**) SDBS contained WS_2_/EG nanofluids and (**d**) CTAB contained WS_2_/EG nanofluids.

Many flow thermal applications operated in a range of temperatures. Therefore, knowledge of the temperature dependent viscosity is important for the process design. The temperature dependent viscosity is presented in the form of relative viscosity from 25 °C to 70 °C, as shown in Figure 11. In the case of the pristine WS_2_/EG nanofluids (Figure 11a), the viscosity was sufficiently below the base fluid. However, an interesting behavior was observed over the entire temperature range. As the temperature increased, the relative viscosity progressively increased, but, overall, it was sufficiently below the base fluid for volume concentrations of up to 0.01%. The higher volume concentration (0.02%) showed the intersecting points close to the benchmark line. Such behavior of nanofluids provides evidence about the critical temperature limit, as shown in Figure 11a. The results revealed that, at a critical temperature, the viscosity inversion occurred, and again it starts increasing. However, in the present case, the higher concentration (0.02%) has shown a critical temperature transition range of between 40 °C and 45 °C. The possible mechanisms behind the existence of a critical temperature could be the fact that, at the same temperature, the base fluid’s viscosity was not reduced sufficiently, due to the viscous hindrance offered by nanoparticles. Thus, such critical temperature behavior could be avoided by using high shear rates at elevated temperatures [31]. Such behavior of nanofluids indicates that the in-depth understanding of temperature dependent viscosity is a vital parameter in deciding the process design parameters, such as the size of pump and the pressure requirements. In the current work, the base fluid viscosity was reduced to ~56.3% and ~74.5% at temperatures of 50 °C and 70 °C, respectively, when compared with the viscosity at 25 °C. Furthermore, the 0.005% WS_2_ NPs’ addition raised this reduction to 64.3% and 75.9%, corresponding to 50 °C and 70 °C, respectively, which can be attributed to the lubrication characteristics of NPs [12]. On the other hand, 0.01% WS_2_ nanofluids improved the viscosity reduction to 58.7% and 75.9%, while 0.02% WS_2_ showed an almost similar trend as the base fluid corresponding to the selected targeted temperatures, respectively. These results suggest the concentration and temperature dependency of dynamic viscosity. In view of the present results, it can be concluded that the lubricating effect of WS_2_ nanoparticles was significant at small concentrations, and it diminished as the concentration increased further from 0.01 vol% to 0.02 vol%. Besides this, the dynamic viscosity reduction potential of WS_2_ NPs was prominent at low temperatures, while along the higher side of the temperature range, the improvement became less prominent. Such behavior transformation of nanofluid viscosity with increasing temperature suggests that, at high temperatures, the base fluid part of the nanofluid governs the viscosity of colloidal systems. Probably, at elevated temperatures, it can also be linked with colloidal stability, as discussed earlier. The primary reason for the reduced viscosity was the breakdown of intermolecular forces among the fluid layers. Such breakdown is associated with the internal structure of nanofluids, which increases with temperature rises due to the enhanced Brownian motion [21].

Moreover, the surfactant-containing nanofluids also showed concentration dependent viscosity behavior, as shown in Figure 11b–d for SDS, SDBS, and CTAB, respectively. Like the oscillation and shear flow behavior, the temperature sweep results for surfactant-containing nanofluids were also similar. However, variation in the viscosity reduction over the temperature scale was flattened for some samples. For instance, as shown in Figure 11b, the SDS surfactant’s addition converted the pristine nanofluids’ behavior, with flat curve formations. This could be due to the colloidal stability of nanofluids which sustains the viscosity reduction due to surfactant micelles up to a certain temperature range. It was also witnessed that the small concentrations of SDS shifted the critical temperature to the higher temperature side for 0.02 vol% WS_2_, whereas the critical temperature range was eliminated at a higher SDS concentration. In addition, 0.01% WS_2_/EG nanofluids clearly revealed that the critical temperature transition was between 45 and 50 °C, while it was absent when no surfactant was used. Intriguingly, the 0.005% WS_2_/EG nanofluids illustrated a synergistic effect with small concentrations of SDS (0.05 vol%), with further improvements in viscous behavior.

The synergistic effect of SDBS and CTAB surfactants on temperature dependent viscosity is also noticeable, as shown in Figure 11c,d, respectively. The SDBS surfactant showed viscosity enhancement at all concentrations and over the entire temperature range, while the CTAB reflected a reduction at certain concentrations. In short, there exist optimum concentrations, which account for the improvements in the flow behavior of nanofluids. Like other studies related to nanofluids, in the present work, the absolute viscosity decreased exponentially with temperature [60]. Finally, a detailed summary of the dynamic viscosity reduction and enhancement at various temperatures is shown in Table 7.

Furthermore, to correlate the experimental temperature dependent viscosity data, an Arrhenius type equation was used [6], as shown in Equation (4).

(4) $$\eta_{eff}=\left(\eta_{\infty ,T}\right)\times \left(e^{\frac{E_{a}}{RT}}\right)$$

where *η_eff__,_* $\eta_{\infty ,T}$*, E_a_*, and *R* are related to the experimental viscosity at certain given temperatures, viscosity at infinite temperature, activation energy, and the universal gas constant, respectively. Both the activation energy and infinite viscosity show the fluid flow behavior. The activation energy terminology was introduced by Savante Arrhenius [61]. Generally, it is the amount of energy needed to transform reactants into products in a chemical reaction. This might be in the form of the potential and kinetic energy of molecules, which could be used for chemical reactions to disentangle their bonds. The random movement of molecules does not initiate a reaction until their momentum is high enough when compared to the base energy barrier. Consequently, the bottom level of energy needed to initiate a chemical reaction is known as activation energy. Here, *E_a_* corresponds to the inter-layer friction in nanofluids. The fitting parameters of the experimental data using Equation (4) are shown in Table 8.

It can be observed from the fitting parameters of the Arrhenius equation that the addition of nanoparticles into the base fluid have radically improved the viscous behavior. The infinite viscosity value decreased, along with a reduction in the activation energy for pristine nanofluids with the addition of NPs. Moreover, it is also notable that, as the concentration of WS_2_ increased, the infinite viscosity displayed significant changes (increased). In short, these findings were well in agreement with the detailed rheological analysis of WS_2_/EG nanofluids. A collective summary based on the stability, thermal conductivity, and rheological analysis, with the combinations of WS_2_/EG nanofluids (having reduced viscosity), is given in Table 9.

## 4. Conclusions

In the present study, a comprehensive experimental evaluation of stability, thermal conductivity, and rheological properties has been carried out. Nanofluids with a wide range of WS_2_ volume concentrations (0.005, 0.01, and 0.02%), along with SDS, SDBS, and CTAB surfactants (0.05, 0.5, 1, and 2%) were characterized for targeted properties in a range of temperatures (25–70 °C). The conclusive remarks as a result of the current experimental investigations are given below.

1. The size and morphology of WS_2_ NPs was confirmed and found to be well in agreement with the supplier data sheet.
2. The addition of SDS (0.05%) increased the zeta potential by ~88% in comparison to 0.005% pristine nanofluid. This rate of improvement reduced to 37% per 0.05% SDS addition when 0.5% SDS was incorporated, but the absolute value of the zeta potential increased. Similarly, for other nanofluid combinations with SDS, the absolute zeta potential values improved, but the rate of improvement with regards to surfactant concentrations became slow as the amount of surfactant increased. In the case of SDS addition, the maximum increment in agglomerate size appeared at ~172%, ~245%, and 261%, corresponding to 0.005% WS_2_ + 2% SDS, 0.01% WS_2_ + 2% SDS, and 0.02% WS_2_ + 0.05% SDS, respectively. Collectively, the zeta potential improved to 554% while the mean particle size also showed an increase up to 411% due to the adsorption of surfactant molecules. This might cause agglomeration with aging, leading to flocculation and sedimentation.
3. The maximum thermal conductivity enhancement was observed to be ~ 2.8%, 1.9%, and 4.5% for combinations of 0.05% SDS + 0.005% WS_2_, corresponding to operating temperatures of 25 °C, 50 °C, and 70 °C, respectively. Subsequently, the increased concentration of SDS decreased the thermal conductivity which is a common observation for higher concentration of surfactants. Like the 0.005% WS_2_, the higher concentrations such as 0.01% WS_2_ and 0.02% WS_2_ also show higher enhancement corresponding to lower surfactant concentrations. However, the elevated temperature behavior showed an oscillating response and the reason may lie within the detachment of surfactant molecules and the interaction with WS_2_ sheets. Therefore, it needs to be explored further. Collectively, the maximum thermal conductivity of pristine nanofluids increased from 3.5% to 6.9% with the addition of surfactants. However, the results also revealed that the maximum thermal conductivity improvement did not correspond to the high zeta potential. Thus, rigorous concentration optimization is always a decisive parameter for the optimum heat transfer fluid solution.
4. The oscillation rheology showed a rational verification of structured network formation inside nanofluids with WS_2_ NPs and also depicted the nanofluids’ behavior transition from viscous to elastic with surfactants. The viscous to elastic structural transition suggested that a higher initial pumping input was required to initiate the fluid flow.
5. The anomalous viscosity reduction of ~8.2% corresponding to the minimum volume concentration (0.005%) of WS_2_ witnessed the super-fluidity of pristine WS_2_/EG nanofluids. In addition, the synergistic effect of small volume concentrations (0.05%) of SDS surfactant was also notable with 0.005% WS_2_, which further reduced the viscosity, and the final reduction became ~10.5%. However, higher concentrations of SDS, SDBS, and CTAB are not beneficial for synergistic effects with nanoparticles, as noted in the present work.
6. All the tested samples revealed a non-Newtonian to Newtonian behavior transition at a shear rate of 10 s^−1^.
7. Particularly for 0.05% SDS with 0.005% WS_2_, thermal conductivity was enhanced by up to 4.5%, with a corresponding decrease in viscosity of up to 10.5%, in a temperature range of 25–70 °C as compared to EG.
8. All in all, the thermal conductivity enhancement up to 6.9% and dynamic viscosity up to 10.5% proposed that the WS_2_/EG nanofluids can be considered as potential candidates for engineering applications. However, WS_2_ based nanofluids have been characterized here for the first time. Therefore, further experimental evaluation is proposed to develop a database of their stability, thermal conductivity, and rheology for comparison purposes.

## Figures and Tables

**Table 1:** Tungsten disulphide (WS_2_) specifications.

Nanoparticles	Purity (%)	Density (g/cm^3^)	Molecular Weight (g/mol)	Average Thickness (nm)	Specific Surface Area (m^2^/g)	Coefficient of Friction	Source
CAS-Reg. No.: 12138-09-9 WS_2_ Silver-Grey Crystalline Solid Powder	99	7.5	248	90	30	0.03–0.07	M K Impex Corp. Mississauga, ON, Canada

**Table 2:** Details of surfactants and base fluid.

Material	Name	Chemical Formula	Molecular Weight (g/mol)	Density (g/cm^3^)	Type (-)	Source
Surfactants	SDS *	NaC_12_H_25_SO_4_	288.38	1.01	Anionic	Fisher Scientific, Loughborough, UK
	SDBS *	C_18_H_29_NaO_3_S	348.48	1.06	Anionic	
	CTAB *	C_19_H_42_BrN	364.45	0.5	Cationic	
Base fluid	EG *	(CH_12_OH)_2_	62.07	1.11	Organic	Sigma Aldrich, Subang Jaya, Malaysia

* SDS: Sodium dodecyl sulphate. SDBS: Sodium dodecylbenzenesulfonate. CTAB: Cetyltrimethylammonium bromide. EG: Ethylene glycol.

**Table 3:** Maximum value of zeta potential for WS_2_/EG nanofluids.

WS_2_ (Vol%)	Surfactant Concentration (vol%)			Maximum Mean Zeta Potential (mV)			
	SDS	SDBS	CTAB	SDS	SDBS	CTAB	No Surfactant
0.005	2	0.5	2	−51.7 ± 4.9	−45.2 ± 2.1	42.3 ± 4.5	−7.9 ± 1.3
0.01	2	1	2	−35.6 ± 5.3	−43.2 ± 2.3	40 ± 2.8	−9.28 ± 1.8
0.02	2	2	2	−35.9 ± 6.8	−42.7 ± 2.3	31.6 ± 3.9	−5.06 ± 0.5

**Table 4:** Optimal surfactant concentrations corresponding to maximum thermal conductivity enhancement at different temperatures.

WS_2_ (Vol%)	Optimal Surfactant Concentration (vol%)			Maximum Thermal Conductivity Enhancement (%)			
	SDS	SDBS	CTAB	SDS	SDBS	CTAB	No Surfactant
*T* = 25 °C							
0.005	0.05	1	0.05	2.8	2.5	2.4	1.6
0.01	0.05	0.05	0.05	1.9	1.5	1.7	1.2
0.02	0.05	0.05	1	2.2	1.7	2	1.2
*T* = 50 °C							
0.005	0.05	0.5	1	1.9	2.1	2.2	−1.4
0.01	0.05	2	2	0.4	2.7	−2.7	−1.6
0.02	0.05	0.05	2	−3.5	1.5	0.4	−1.9
*T* = 70 °C							
0.005	0.05	0.5	0.5	4.5	1.3	4.1	3.9
0.01	2	0.5	1	4.6	1	1.3	3.5
0.02	2	0.05	0.05	3.0	1.7	6.9	0.4

**Table 5:** Strain sweep results summary on WS_2_/EG nanofluids (continued).

Sample Description	Flow Point Stress (mPa) at G’ = G”	Resulting Remarks
Base fluid (EG)	No crossover	Very high loss factor with high fluidity over entire deformation range
0.005 vol% WS_2_	No crossover	G’ increased as compared to EG but still high fluidity over entire deformation range
0.01 vol% WS_2_	No crossover	
0.02 vol% WS_2_	No crossover	
0.005 vol% Ws_2_ + 0.05 vol% SDS	4.2	Behaves strongly gel-like until 1.218% of deformation
0.01 vol% Ws_2_ + 2 vol% SDS	No significant crossover	Oscillatory behavior followed by completely liquid-like over high deformation range
0.02 vol% WS_2_ + 0.05 vol% SDS	No significant crossover	Oscillatory behavior followed by completely liquid-like over high deformation range
0.02 vol% WS_2_ + 2 vol% SDS	No significant crossover	Weak gel behavior at low deformation followed by liquid behavior over high deformation range
0.005 vol% Ws_2_ + 1 vol% SDBS	3.548	Behaves like weak gel until 1.646% of deformation
0.01 vol% Ws_2_ + 2 vol% SDBS	9.05	Strong gel until 2.779% of deformation
0.02 vol% Ws_2_ + 0.05 vol% SDBS	No significant crossover	Weak gel behavior at low deformation followed by liquid behavior over high deformation range
0.005 vol% Ws_2_ + 0.5 vol% SDBS	No crossover	Very high loss factor with high fluidity over entire deformation range
0.02 vol% Ws_2_ + 0.05 vol% CTAB	8.289	Weak gel until 1.095% of deformation
0.005 vol% Ws_2_ + 0.5 vol% CTAB	2.597	Weak gel until 0.5343% of deformation
0.005 vol% Ws_2_ + 1 vol% CTAB	3.815	Weak gel until 1.32% of deformation
0.005 vol% Ws_2_ + 0.05 vol% CTAB	No crossover	Very high loss factor with high fluidity over entire deformation range

**Table 6:** Herschel–Bulkley and Bingham model fitting parameters.

Sample Description	H–B Model				Bingham Model		
	*τ_o_* (mPa)	*K* (mPa.s*^n^*)	*n* (-)	*R* ^2^	*τ_o_* (mPa)	*μ* (mPa.s)	*R* ^2^
Base fluid (EG)	1.5421	14.575	1.0013	0.99999	1.0866	14.658	0.99999
0.005 vol% WS_2_	1.6031	13.258	1.0034	0.99999	0.50148	13.46	0.99999
0.01 vol% WS_2_	−0.77283 *	13.735	0.99937	0.99999	−0.56718 *	13.697	0.99999
0.02 vol% WS_2_	1.4844	14.247	1.0018	0.99999	0.85551	14.363	0.99999
0.005 vol% WS_2_ + 0.05 vol% SDS	3.4724	12.812	1.0052	0.99999	1.8621	13.106	0.99999
0.01 vol% WS_2_ + 2 vol% SDS	−0.49429 *	14.836	0.99817	0.99999	0.14732	14.718	0.99999
0.02 vol% WS_2_ + 0.05 vol% SDS	−1.3374 *	14.487	0.99677	1	−0.23628 *	14.284	1
0.02 vol% WS_2_ + 2 vol% SDS	−2.0203 *	15.367	0.99483	0.99999	−0.16437 *	15.023	1
0.005 vol% WS_2_ + 1 vol% SDBS	−1.1213 *	15.57	0.99524	0.9998	0.61465	15.249	0.99998
0.01 vol% WS_2_ + 2 vol% SDBS	−2.2094 *	16.01	0.99384	0.99999	0.090188	15.584	0.99999
0.02 vol% WS_2_ + 0.05 vol% SDBS	3.9121	14.659	1.0064	0.99998	1.6365	15.074	0.99999
0.005 vol% WS_2_ + 0.5 vol% SDBS	0.18404	15.014	0.99904	1	0.52664	14.951	1
0.02 vol% WS_2_+ 0.05 vol% CTAB	13.844	15.654	0.9931	0.99996	16.393	15.186	0.99995
0.005 vol% WS_2_ + 0.5 vol% CTAB	1.6446	14.449	0.99751	0.99999	2.4965	14.292	0.99998
0.005 vol% WS_2_ + 1 vol% CTAB	15.344	13.99	1.01	0.99997	11.846	14.62	0.99998
0.005 vol% WS_2_ + 0.05 vol% CTAB	−3.02 *	15.146	0.99256	0.99998	−0.41809 *	14.66	0.99998

* Yield stress cannot be calculated.

**Table 7:** Comparison of viscosity data for enhancement and reduction (%) at targeted temperatures of 25 °C, 50 °C, and 70 °C, using a constant shear rate of 50 s^−1^. “+” sign indicates enhancement and “–” sign indicates reduction in viscosity as compared to the base fluid.

Sample Description	Viscosity Enhancement/Reduction (%)		
	25 °C	50 °C	70 °C
0.005 vol% WS_2_	−8.2	−6	−4.7
0.01 vol% WS_2_	−6.9	−5.3	−4
0.02 vol% WS_2_	−2.1	−0.2	−1
0.005 vol% WS_2_ + 0.05 vol% SDS	−10.5	−8.1	−5.6
0.01 vol% WS_2_ + 2 vol% SDS	0.11	4.9	3
0.02 vol% WS_2_ + 0.05 vol% SDS	−3.1	−2.8	−0.6
0.02 vol% WS_2_ + 2 vol% SDS	2.1	3.2	1.9
0.005 vol% WS_2_ + 1 vol% SDBS	3.3	3	1.9
0.01 vol% WS_2_ + 2 vol% SDBS	5.7	6.4	6.7
0.02 vol% WS_2_ + 0.05 vol% SDBS	3.1	3	2
0.005 vol% WS_2_ + 0.5 vol% SDBS	1.5	1.4	0.8
0.02 vol% WS_2_ + 0.05 vol% CTAB	4.9	3.6	2.9
0.005 vol% WS_2_ + 0.5 vol% CTAB	−2.3	−2.3	−2.7
0.005 vol% WS_2_ + 1 vol% CTAB	1.8	−0.9	1
0.005 vol% WS_2_ + 0.05 vol% CTAB	−0.7	−0.7	−0.8

**Table 8:** Arrhenius equation fitting parameters for WS_2_/EG nanofluids.

Sample Description	Arrhenius Equation Fitting Parameters		
	$\eta_{\infty ,T}\;(\mathrm{mPa}.s)$	*E_a_* (Jmol^−1^)	*R* ^2^
Base fluid (EG)	33.33762	0.27004	0.99723
0.005 vol% WS_2_	29.80826	0.2618	0.99712
0.01 vol% WS_2_	30.40985	0.26392	0.99712
0.02 vol% WS_2_	32.17606	0.26519	0.99796
0.005 vol% WS_2_ + 0.05 vol% SDS	28.97151	0.26066	0.9968
0.01 vol% WS_2_ + 2 vol% SDS	32.46486	0.26107	0.99781
0.02 vol% WS_2_ + 0.05 vol% SDS	32.11054	0.26817	0.99685
0.02 vol% WS_2_ + 2 vol% SDS	33.89205	0.26831	0.99794
0.005 vol% WS_2_ + 1 vol% SDBS	34.6949	0.2721	0.99738
0.01 vol% WS_2_ + 2 vol% SDBS	34.98507	0.26773	0.99721
0.02 vol% WS_2_ + 0.05 vol% SDBS	34.60862	0.27182	0.9976
0.005 vol% WS_2_ + 0.5 vol% SDBS	33.89232	0.27049	0.99729
0.02 vol% WS_2_ + 0.05 vol% CTAB	35.27397	0.27409	0.99646
0.005 vol% WS_2_ + 0.5 vol% CTAB	32.54759	0.26995	0.99732
0.005 vol% WS_2_ + 1 vol% CTAB	34.31033	0.27608	0.99499
0.005 vol% WS_2_ + 0.05 vol% CTAB	33.16464	0.27049	0.9973

**Table 9:** Parametric comparison of current findings.

Sample Description	Thermal Conductivity Enhancement/Reduction			Viscosity Reduction			Particle Size	Zeta Potential	
	(%)			(%)			(nm)	(−mV)	
	25 °C	50 °C	70 °C	25 °C	50 °C	70 °C	25 °C		
0.005 vol% WS_2_	1.6	−1.4	3.9	−8.2	−6	−4.7	378.28 ± 34.3		7.9 ± 1.3
0.01 vol% WS_2_	1.2	−1.6	3.5	−6.9	−5.3	−4	335.81 ± 38.9		9.28 ± 1.8
0.02 vol% WS_2_	1.2	−1.9	0.4	−2.1	−0.2	−1	388.8 ± 25.7		5.06 ± 0.5
0.005 vol% WS_2_ + 0.05 vol% SDS	2.8	1.9	4.5	−10.5	−8.1	−5.6	749.2 ± 50.1		14.9 ± 1.5
0.02 vol% WS_2_ + 0.05 vol% SDS	2.2	−3.5	3	−3.1	−2.8	−0.6	1224.6 ± 65.3		8.7 ± 1.1
0.005 vol% WS_2_ + 0.5 vol% CTAB	2.3	0.2	4.1	−2.3	−2.3	−2.7	532.8 ± 28.4		−22 ± 2.6
0.005 vol% WS_2_ + 0.05 vol% CTAB	2.4	−0.6	−2.4	−0.7	−0.7	−0.8	338.4 ± 10.2		5.8 ± 0.5

## Supplementary Materials

The following are available online at https://www.mdpi.com/2079-4991/10/7/1340/s1, Table S1: Mean thermal conductivity data with 95% confidence interval, Figure S1: Effect of surfactant on the thermal conductivity of base fluid, Figure S2: Amplitude sweep results of WS_2_/EG nanofluids containing SDS surfactant with less significant elastic domain, Figure S3: Amplitude sweep results of WS_2_/EG nanofluids containing SDBS surfactant with less significant elastic domain, Figure S4: Amplitude sweep results of WS_2_/EG nanofluids containing CTAB surfactant with less significant elastic domain.
