# Supplementary material for: Two-Dimensional Tungsten Disulfide-Based Ethylene Glycol Nanofluids: Stability, Thermal Conductivity, and Rheological Properties
Source: Nanomaterials (Basel). 2020 Jul 9;10(7):1340. doi: 10.3390/nano10071340 (PMC7408399; doi:10.3390/nano10071340)
Supplement: Supplementary file 1 [file nanomaterials-10-01340-s001.pdf]

Table S1. Mean thermal conductivity data with 95% confidence interval.

Surfactant (vol %)		WS_2 0.005 vol%			WS_2 0.01 vol%			WS_2 0.02 vol%			EG		
		25 °C	50 °C	70 °C	25 °C	50 °C	70 °C	25 °C	50 °C	70 °C	25 °C	50 °C	70 °C
		Mean thermal Conductivity (W/m.K), number of measurements (*n*) = 10											
**SDS**	0	0.2522±0.0006	0.2556±0.0018	0.2701±0.0126	0.2513±0.0004	0.2551±0.0056	0.2689±0.0146	0.2513±0.0007	0.2543±0.0044	0.261±0.008	0.2482±0.0016	0.2593±0.0077	0.2599±0.0182
	0.05	0.2552±0.0009	0.2642±0.0072	0.2716±0.0161	0.2529±0.0014	0.2604±0.0044	0.2604±0.0100	0.2537±0.0007	0.2503±0.0022	0.2651±0.0146	0.2493±0.0023	0.2557±0.0031	0.2574±0.0013
	0.5	0.2541±0.0003	0.263±0.008	0.2616±0.0083	0.2521±0.0017	0.2583±0.0071	0.2627±0.0090	0.2521±0.0012	0.2582±0.0021	0.2648±0.0103	0.250±0.0075	0.2585±0.0041	0.2588±0.0031
	1	0.2528±0.0014	0.2621±0.0073	0.2702±0.0137	0.2524±0.0009	0.2598±0.0045	0.2601±0.0054	0.2518±0.0009	0.2533±0.0041	0.2651±0.0104	0.2499±0.0017	0.2547±0.0032	0.2575±0.0030
	2	0.2531±0.0009	0.2548±0.0029	0.2701±0.0124	0.2509±0.0009	0.2522±0.0067	0.2718±0.0166	0.2503±0.0009	0.2575±0.0053	0.2676±0.0115	0.2492±0.0013	0.2528±0.0033	0.2471±0.0049
SDBS	0.05	0.2523±0.0005	0.2487±0.0036	0.2573±0.0072	0.252±0.002	0.2548±0.0047	0.259±0.006	0.2524±0.0005	0.2633±0.0059	0.2643±0.0053	0.2491±0.0011	0.2541±0.0024	0.2533±0.0065
	0.5	0.253±0.002	0.2647±0.0131	0.2633±0.0123	0.2496±0.0011	0.2608±0.0082	0.2625±0.0099	0.2516±0.0008	0.2628±0.0071	0.2629±0.0054	0.2498±0.0027	0.2546±0.0034	0.2586±0.0083
	1	0.2544±0.0005	0.261±0.006	0.2565±0.0063	0.2489±0.0016	0.2659±0.0105	0.261±0.017	0.2509±0.0006	0.2624±0.0046	0.2627±0.0042	0.2499±0.0021	0.2539±0.0024	0.2594±0.0114
	2	0.2523±0.0019	0.2559±0.0054	0.2613±0.0084	0.2506±0.0013	0.2663±0.0045	0.2523±0.0045	0.2488±0.0009	0.2598±0.0071	0.2508±0.0071	0.2507±0.0009	0.2518±0.0026	0.2544±0.0057
CTAB	0.05	0.2541±0.0009	0.2578±0.0042	0.2539±0.0055	0.2525±0.0008	0.2537±0.0028	0.2552±0.0093	0.2492±0.0012	0.2546±0.0063	0.2778±0.0259	0.2502±0.0018	0.2587±0.0045	0.2554±0.0169
	0.5	0.2539±0.0009	0.2598±0.0053	0.2705±0.0158	0.2525±0.0007	0.2556±0.0038	0.2579±0.0097	0.2518±0.0010	0.2525±0.0031	0.2613±0.0091	0.2495±0.0020	0.2512±0.0019	0.2499±0.0051
	1	0.2536±0.0007	0.265±0.005	0.2496±0.0072	0.2513±0.0008	0.2557±0.0052	0.2633±0.0128	0.2532±0.0006	0.2541±0.0040	0.2537±0.0066	0.2492±0.0015	0.2522±0.0034	0.256±0.0125
	2	0.2500±0.0006	0.2569±0.0043	0.2429±0.0101	0.2501±0.0007	0.2523±0.0025	0.2634±0.0128	0.2521±0.0008	0.2603±0.0037	0.2679±0.0074	0.2493±0.0020	0.2584±0.0044	0.2567±0.0173

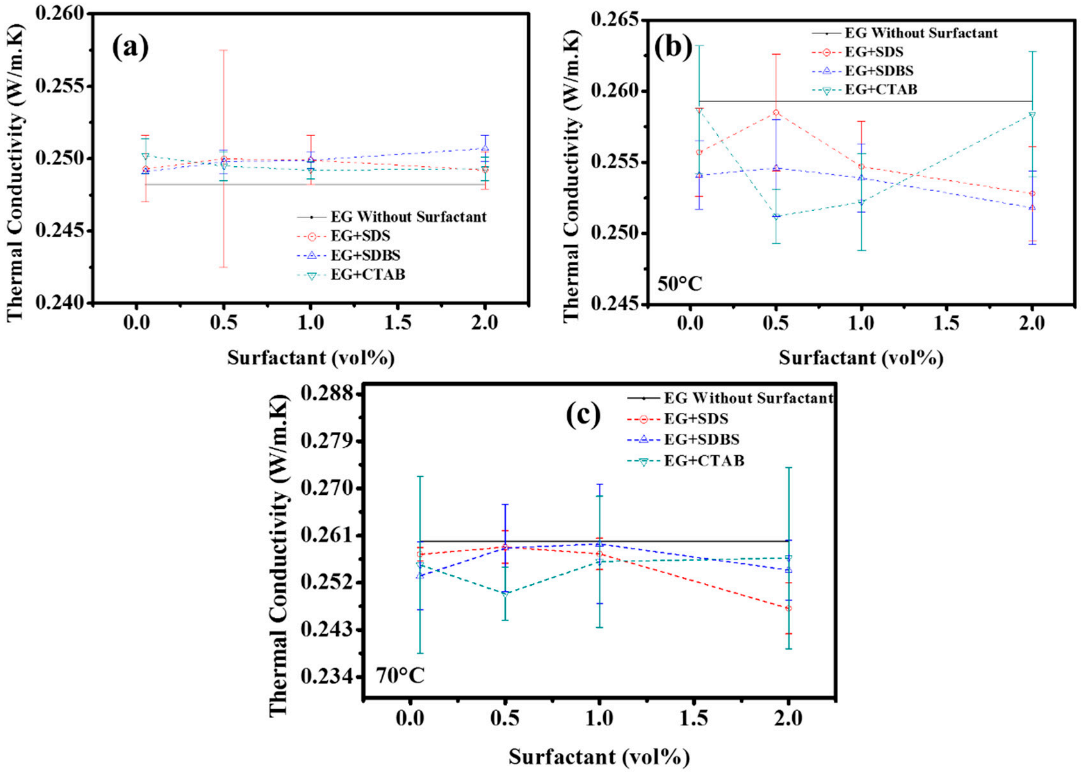

Figure S1. Effect of surfactant on the thermal conductivity of base fluid: **a**) at 25 °C, **b**) at 50 °C and **c**) at 70 °C.

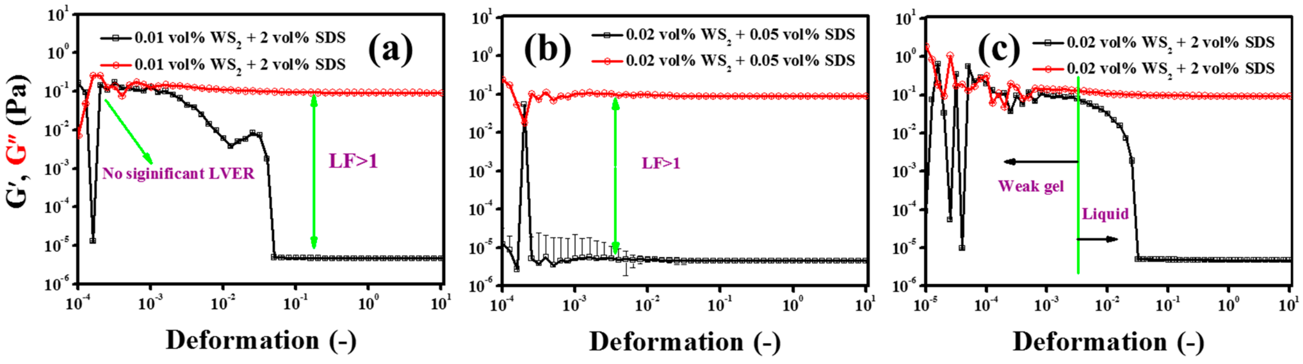

Figure S2. Amplitude sweep results of WS_2/EG nanofluids containing SDS surfactant with less significant elastic domain: **a**) 0.01 vol% WS_2 + 2 vol% SDS, **b**) 0.02 vol% WS_2 + 0.05 vol% SDS and **c**) 0.02 vol% WS_2 + 2 vol% SDS.

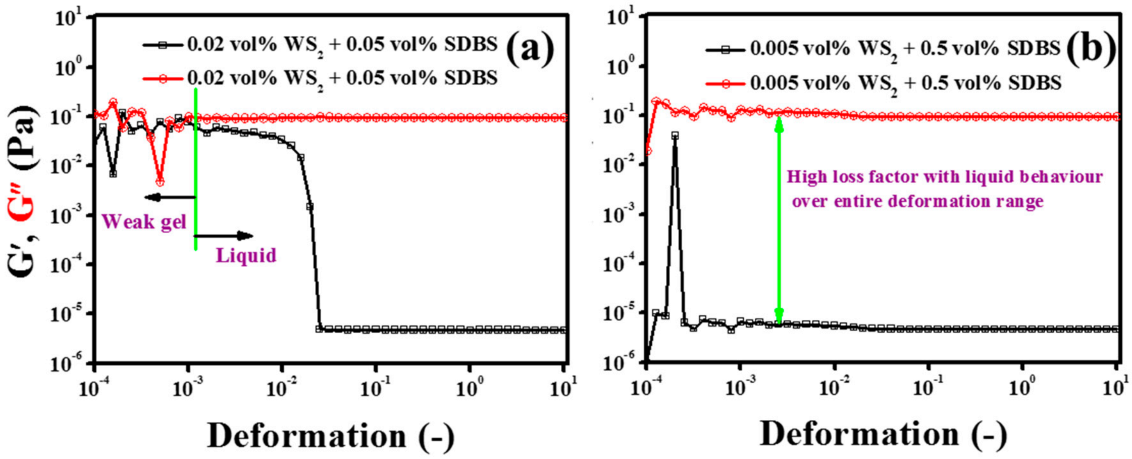

Figure S3. Amplitude sweep results of WS_2/EG nanofluids containing SDBS surfactant with less significant elastic domain: **a**) 0.02 vol% WS_2 + 0.05 vol% SDBS and **b**) 0.005 vol% WS_2 + 0.5 vol% SDBS

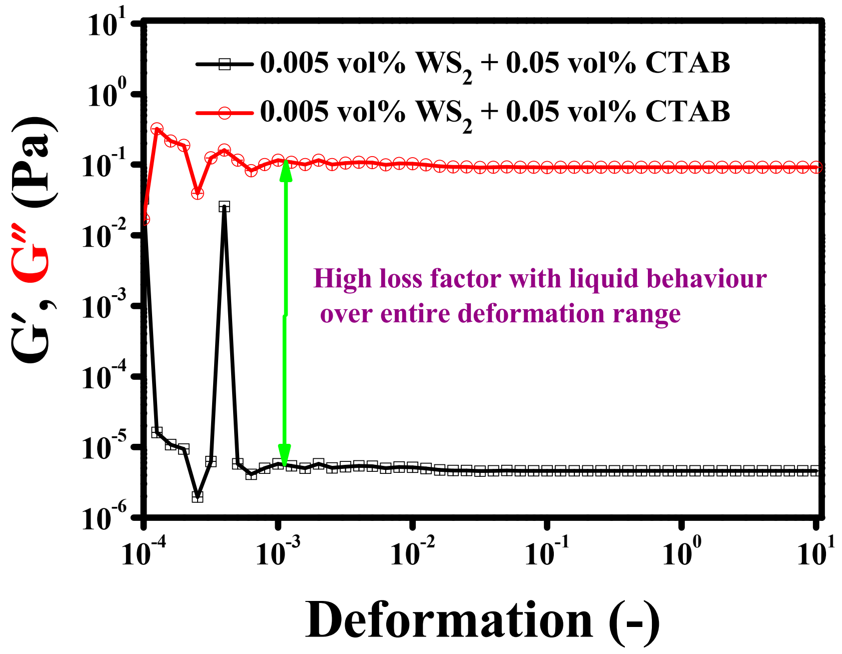

Figure S4. Amplitude sweep results of WS_2/EG nanofluids containing CTAB surfactant with less significant elastic domain.
